# The case of Iranian immigrants in the greater Toronto area: a qualitative study

**DOI:** 10.1186/1475-9276-11-9

**Published:** 2012-02-27

**Authors:** Mahdieh Dastjerdi

**Affiliations:** 1 Faculty of Health, School of Nursing, York University, Toronto, ON, Canada

**Keywords:** Access to health care, Iranian immigrants and refugees, Canada, GTA, Toronto, Health care providers, Health care professionals, Social workers

## Abstract

**Introduction:**

Iranians comprise an immigrant group that has a very different cultural background from that of the mainstream Canadian population and speaks a language other than English or French; in this case mainly Farsi (Persian). Although Iranian immigrants in Toronto receive a high proportion of care from Farsi-speaking family physicians and health care providers than physicians who cannot speak Farsi, they are still not satisfied with the provided services. The purpose of this study was to identify the obstacles and issues Iranian immigrants faced in accessing health care services as seen through the eyes of Iranian health care professionals/providers and social workers working in Greater Toronto Area, Canada.

**Methods:**

Narrative inquiry was used to capture and understand the obstacles this immigrant population faces when accessing health care services, through the lens of fifty Iranian health care professionals/providers and social workers. Thirty three health care professionals and five social workers were interviewed. To capture the essence of issues, individual interviews were followed by three focus groups consisting of three health care professionals and one social worker in each group.

**Results:**

Three major themes emerged from the study: language barrier and the lack of knowledge of Canadian health care services/systems; lack of trust in Canadian health care services due to financial limitations and fear of disclosure; and somatization and needs for psychological supports.

**Conclusion:**

Iranians may not be satisfied with the Canadian health care services due to a lack of knowledge of the system, as well as cultural differences when seeking care, such as fear of disclosure, discrimination, and mistrust of primary care. To attain equitable, adequate, and effective access to health care services, immigrants need to be educated and informed about the Canadian health care system and services it provides. It would be of great benefit to this population to hold workshops on health topics, and mental health issues, build strong ties with the community by increasing their involvement, use plain language, design informative and health related websites in both Farsi and English, and provide a Farsi speaking telephone help line to answer their health related issues.

## Introduction

Canada is home to people of various cultural groups who speak languages other than English or French. Iranians comprise an immigrant group that has a very different cultural background from that of the mainstream Canadian population and speaks a language other than English or French; in this case mainly Farsi (Persian). The Greater Toronto Area (GTA) is the largest metropolitan area in Canada, with 5.5 million populations. The GTA is usually defined as the central city of Toronto, along with four regional municipalities surrounding such as Durham, Halton, Peel, and York. The city of Toronto accounted for 22.9% of all visible minority persons in Canada, and 42.4% of visible minority person in Ontario. More than 200 distinct ethnic origins residents identified in Toronto. Almost 1 in 4 visible minority persons in Canada resides in Toronto. Within the GTA, the city of Toronto had 58.8% of visible minority persons. Other cities such as Peel region was home to a large number of visible minority persons was 26% followed by York region 10.2%, Halton Region 2.9%, and Durham Region 2.2% [1,2].

Farsi (Persian) language ranked 9 of the top ten home languages 1.2% [1,2]. There are more than 95,420 Iranians in Canada (both permanent residents and non-permanent residents) and Iran was one of the top ten source countries for permanent residents in Canada in the past years (2006-2008) [1]. According to official statistics, there are more than 40,000 Iranians in the GTA [2]. Immigrant populations born outside of Canada including refugees, legal and illegal residents, may experience health disparities. It is well documented that the health status of immigrants at the time of arrival is high and it declines and converges after about five years compared to the native-born population [3-8].

Much research regarding the health of immigrants has concentrated on psychiatric issues, such as post-traumatic stress disorder (PTSD), depression, mental disorders, mental health, suicide, stress, and adaptation or coping [9-11]. Other researchers have studied acculturation, assimilation, concepts of health, health status, and lack of cultural competence [12,13]. Lasser, Himmelstein, and Woolhandler performed a comparative study about access to care, health status, and utilization of medical services according to race, income, and immigration status in the United States and Canada. They found that US residents are one third less likely to have regular family physicians, one fourth more likely to have unmet health care needs, and more than twice as likely to need medications. Health disparities were presented on the basis of race, income and immigration status in both countries, but were more pronounced in the United States [14]. The study did not single out which ethnic groups with what historical backgrounds experienced more unmet health care needs and risk factors in health disparities.

Although only a few studies focused on the use of health services by Iranian immigrants or refugees, some issues pertained more directly to the culture of the host country. One issue found in all studies, regardless of host country, was the problem of language differences and communication barriers [15-20]. Dastjerdi examined the process by which Iranian immigrants learn to access Canadian health care services in Edmonton, Alberta. Findings suggest that effectiveness, responsiveness, and appropriateness of services play an important role in using healthcare services persistently [20].

Studies on ethnic matching showed that matching clients from a minority group with clinicians from the same ethnic background enhanced the use of community mental health services, led to the declining use of emergency services and the delaying use of mental health services [21-24]. Health care is a social process in which both the health care providers and clients bring a set of beliefs, expectations, culture, and practices to the encounter. Although it is recommended that health care providers from the same community as their clients facilitate access to health care services for those clients, immigrants may still not follow preventive care and treatment regimens. Through careful observations, reading local Farsi publications, and through personal conversations, I found that Iranians in the GTA have more access to Iranian health care professionals/providers than Iranians in Edmonton, but they are still not satisfied with the provided services. Therefore, it is worth scrutinizing this issue and pinpointing obstacles of access to health care services as seen through the lens of health care providers and social workers who work with Iranian immigrants.

In the GTA, Iranian immigrants receive a high proportion of care from Farsi-speaking family physicians and health care providers than physicians who cannot speak Farsi (Persian). The purpose of this study was to identify the obstacles and issues Iranian immigrants faced in accessing health care services as seen through the eyes of Iranian health care professionals and social workers working in Greater Toronto Area, Canada.

## Methods

Narrative inquiry is located within the interpretive paradigm of human science research, and it contributes to our understanding of the perceptions of the research participants and their life world. Narrative inquiry is the process of gathering information for the purpose of research through storytelling [25]. Connelly and Clandinin stated that, "Humans are storytelling organisms who individually and collectively lead storied lives. Thus, the study of narrative is the study of the ways humans experience the world (p.2)." [26]. In other words, people's lives consist of stories. According to Morse, purposive sampling requires deliberate selection of participants who have "experiential fit" [27]. Participants were recruited in GTA through community health, social service agencies, and word of mouth using purposive sampling with the 87% response rate.

I used narrative inquiry to capture and understand the obstacles this immigrant population faces when accessing health care services, through the lens of fifty Iranian health care professionals/providers (HCP) and social workers (SW), all of whom work with Iranians living in the GTA (Table 1). Social workers were chosen because they usually have a better understanding of the complexity of the communities, and immigrants' settlement struggles. I conducted in-depth semi-structured individual interviews with 33 health care professionals, and five social workers respectively. To capture the essence of issues, individual interviews were followed by three focus groups (FG) consisting of three health care professionals and one social worker in each group. The interview started with a very broad opening statement, such as "Tell me more about the most challenging experience in working with Iranian immigrant clients" and "In your opinion, what kind of barriers immigrants face in accessing healthcare services". The study received approval from York University Ethics Board.

**Table 1:** Demographic Data

Profession	Educated solely in Canada*	Educated in Iran and had some training in Canada*	Years of experience in Canada	Total
Physician	3	12	3 to 15 years	15

Registered Nurse	4	7	2 to 6 years	11

Psychotherapist/ Psychologist	3	2	1 to 8 years	5

Dentist	2	4	4 to 10 years	6

Pharmacist	1	4	3 to 10 years	5

Social worker	6	2	2 to 15 years	8

Total	20	30	1 to 15 Years	50

## Results

Three major themes emerged from the study: language barrier and the lack of knowledge of Canadian health care services/systems; lack of trust in Canadian health care services due to financial limitations and fear of disclosure; and somatization and needs for psychological supports.

### Language barrier and lack of knowledge of the Canadian health care system

Many non-English speaking newcomers, in general, find communication to be an important obstacle to living in a new country and to accessing health care services. A study of Iranian immigrants living in Edmonton showed that upon their arrival, they were provided many forms and pamphlets about living in Canada in English, French, and other languages that they did not understand. Those who could read English or French found the pamphlets hard to follow, long, in small print and inconvenient. Consequently, they put them away without going through all the pages. Although they received some information in Farsi, for cultural reasons they did not consider information written on a piece of paper seriously [20].

In this study, Iranian health care providers indicated that although language barrier was an issue for Iranian immigrants in Canada, ethnic matching helped these patients overcome communication and cultural differences to some extent. They emphasized that most of the newcomers had limited knowledge of the host country's health care services. The health care professionals pointed out that health literacy and cultural understanding of the Canadian health care system and services by immigrants remained outstanding.

Almost all the participants agreed that language deficit poses a health care barrier for these patients. One of the health care professionals considered language barrier as a two-sided issue.

"For some health care providers like me, Farsi is our second language in the profession not used on a daily basis. Since we got our education in English, we don't know how to translate some phrases to Farsi. We don't understand some slang or Farsi idioms related to health. Therefore, it is health care providers who cannot understand or communicate with them. The language barrier is for both sides, both sides could run into problems." (HCP # 5)

Apart from language barrier, some participants believed that lack of knowledge of the Canadian health services might lead health care consumers to delay using provided services effectively.

"Since I can speak the same language as my clients, language is not an issue. There are still numbers of issues in regards to making decisions about treatment, and following up necessary advice. Most of our patients don't know how the Canadian health care system runs. They expect us to manage their problems as we do in Iran. As a result, sometimes they are not happy with our methods of practice." (HCP # 7)

"The most challenging part is to educate them about what is right and what is wrong regarding their issues. They understand the system differently, as a result they follow it differently. They do not accept the definition of terms." (SW # 4)

"They are scared. They have a vague idea about reporting child abuse. They are afraid, so when they come to me they don't say much. Because they say, "we've heard they will come and take our kids away". Again, it shows lack of knowledge ... as a result, they cannot use services properly. They don't know what's right [correct], what's wrong [incorrect]." (HCP # 3)

### Lack of trust in Canadian health care services

Trust is a multilayered concept, and it can be influenced in many ways. Most participants believed that their clients were skeptical and unable to trust the services and never felt completely comfortable using them.

*"Some patients do not trust Canadian health care services due to long waiting hours, not having access to their lab reports, and not having direct access to specialists. Some of my patients ask me to refer them to specialists. They like to choose their specialists and shop around to find the one they can trust, as they do in Iran." (HCP # 10)*

Some mentioned that a few Iranians preferred to visit non-Iranian physicians or social workers in order to keep their personal life private and secure.

"Since our community is not large, and people know each other, they are reluctant to talk about their limitations and personal issues. Despite a language barrier, in order to keep their face and honor they prefer to have non-Iranian health care providers or social workers." (SW # 2)

In addition, a participant mentioned that the unique culture of an individual health consumer played an important role in their ability to establish trust in providers and services.

"When it comes to some issues, such as violence or committing suicide, we have to help them and report the incident. We have to support and protect them. In this case, they get angry at us. I had one patient who was stressed out and crying a lot. After a two-hour meeting, she was sobbing and said that "as soon as I leave your office, I will kill myself". Many times, I asked her many times about the seriousness of her statement. Each time she nodded "yes". I reported the suicide. She got mad at me and said, "the big mistake I made was trusting you, and I'll never do it again." (HCP # 3)

Some participants pointed out that their clients had unrealistic expectations that led to dissatisfaction and mistrust of health care providers and services.

"Sometimes, they called me in the middle of the night and asked me what to do for their kids. My area of practice is different. When I told them, I couldn't do anything, but they could ask help from such and such.... and I would be there to help them. They got upset and they thought I could do something, but I didn't want to help them. That is very frustrating and heart breaking." (HCP # 17)

In this study, clients' financial limitations and looking for shortcuts to services or alternatives were identified as factors that affected trust. Participants mentioned that few immigrant families could afford the cost of treatment such as medications and some special medical tests, eye care, dental care and the like. Without having a stable well-paid job, they could not afford to buy private insurance.

"As a social worker, I face many challenges every day. We have limited resources available to help people in need. The biggest and the most frustrating part is the paper work. The process takes a long time and clients lose their interest. They think that we do not take them seriously. I know some doctors, dentists, and optometrists who are willing to help clients with limited incomes. Still, it is not enough. We need more help and support." (SW # 1)

Considering the lack of knowledge of the health care system and services, mistrust and the need to save face and hold on to their honor, many of these clients looked for shortcuts and alternatives such as sharing medications, self treatment, and using emergency services rather than visiting family physicians.

"They ask for short cuts. They ask us to show them how they can cut the costs. I do my best to show them what is available, but as you know there are few things that we can do to help them." (HCP # 20)

Confidentiality is very important when it comes to disclosure, and plays an essential role in building and maintaining trust between health care providers and their patients. The Iranian community is small and people may recognize and see each other often. Therefore, this situation makes them hesitant to reveal sensitive issues to the health care professionals. In this study, participants believed that clients' fear of disclosure was a contributing factor to mistrust of the system.

"Our community is a small community. People know each other somehow. We meet each other on different occasions. We grew up in a hush-hush country, and we are very cautious about sharing personal issues. They worry that their stories may leak out to the community. They feel threatened." (FG # 1)

Some participants pointed out that in addition to confidentiality and fear of disclosure, some clients had difficulties differentiating between what was private and public information.

"They think whatever happens in their house, behind closed doors, should stay private. If spousal or child abuse happens in the home, they believe it should stay private. The most frustrating issue that I have with Iranians is the mandatory report that I have to do. They do not trust me. I explain to them that it is for their own good. They don't accept it. They hate me. They think that I am their enemy." (HCP # 3)

It was notable that in spite of having previous bad experiences regarding trust, these clients tried to create a safe space for communication.

"Making decisions about what, how and how much to say is hard. Since they are very selective in disclosing events in their lives, the experience causes many pressures. They have different lives; they share things with their parents but not with partners or their children; they share things with their partner that they never share with their families. They are more comfortable in disclosing their issues to their close friends who often live in Iran or another country. They try to create a safe space and see the disclosure to providers as a threat to it." (FG # 2)

### Somatization and needs for psychological support

All the above-mentioned issues are interwoven and may lead to psychological stress and mental disorders. The stressors related to adjusting to a new country, new culture, new language, and new job are very common for most immigrants. One of the psychological stress manifestations is somatization. Most participants noted that their patients were overwhelmed, and under a great deal of psychological stress--they complained of chest pain, headache, and other physical symptoms leading to a negative impact on overall well-being.

"They complain of having chest pain, headache, insomnia, and lack of concentration. Consequently, they drop courses, take leave from jobs and don't show up for appointments and meetings. They do not believe in our practice. They think that they are really sick physically and they need treatment and some days off work. This situation puts practitioners in a difficult position." (HCP # 27)

Although somatization is considered a defense mechanism, it can lead to severe mental and psychological disorders. These patients need timely care and treatment with serious consideration.

"Due to bearing a lot of stress, they develop anxiety and possibly become isolated. Meanwhile, when they come to our office and talk about their problems, I find that most of their symptoms are related to their psychological status and this puts their mental health at risk. When educated as to what is going on, they invariably disagree with the diagnosis, and ultimately won't accept the advised method of treatment." (FG # 3)

Because they (immigrants) wanted to build a good record of Canadian experience, and keep their jobs, they were hesitant to ask for sick leave. This situation led them to delay or avoid medical follow up. They felt they were trapped.

"They tell me that even if they accept my diagnosis and visit a psychologist, it will create more problems for them. They say, "I have no coverage for that, and I cannot afford it. Moreover, my manager thinks that I have some mental issues, and I might lose my job. It goes on my record, and it will have a negative effect. After that, whatever happens will be judged based on my psychological status". After listening to them, I believe I would think and feel the same way." (HCP # 30)

Clearly, it is not entirely about immigrants accepting their health issues--it is also about providing a support system and helping them to gain control over their lives in a new country.

## Discussion

The purpose of this study was to identify the obstacles and issues Iranian immigrants faced in accessing health care services as seen through the eyes of Iranian health care professionals and social workers in Greater Toronto Area, Canada. Three major themes emerged from the study: language barrier and lack of knowledge of Canadian health care services/systems; lack of trust in Canadian health care services due to financial limitations and fear of disclosure; and somatization and needs for psychological support.

In this study, all participants were immigrants, bilingual, and identified themselves as Iranian- Canadian. They were familiar with their patients' culture, sensitive to their values, norms and expectations. Although the educational background of each participant was different, they shared some noteworthy similarities. More than half of the participants completed their education in Iran, and received their professional designation in Canada and were therefore familiar with the Canadian health care services. Although participants had different years of experience and specialties, because of their similarities, it can be assumed that these participants shared similar experiences in working with Iranian immigrants or refugees in Canada. In addition, since all participants were immigrants themselves, they would have experienced the similar challenges as their clients in the adjustment process.

The results of this study are aligned with an earlier study of Iranian immigrant populations and their access to health care services [20]. Although language barrier has been recognized as a primary issue for immigrants in many studies [6,7,14,15,17-21,28,29], this study interestingly showed that language barrier can be considered a two-sided issue. Some health care professionals mentioned although they knew Farsi they had difficulties to understand some words or context. Their knowledge of Farsi was not as good as their clients. In addition, this study showed that although hiring trained interpreters and cultural brokers, as well as ethnically matched health care providers answered some of the issues around accessing health services, these clients were still not satisfied with the Canadian health care services. Understanding of the health care system and using provided services are culturally constructed. Iranian immigrants' world view, social structures, language, and cultural values influenced their health beliefs, practices, and attitudes [30]. Issues contributing to the health disparities of immigrants were cultural differences in health care seeking patterns and the differences in the perception of health care services [31]. Culture shapes an individual's perception of health, illness, and compliance with diagnosis and treatment regimens. Health culture is communicated through social networks and interaction with others--it is the way in which people learn to interpret health issues and how to react appropriately [32]. This study revealed that, although health care providers and consumers shared the same language and were from the same country, patterns of misunderstandings were still found between them due to cultural misunderstandings and lack of knowledge of the health care system and services. More importantly, lack of knowledge of the health care services led to dissatisfaction and mistrust.

Trust is a multilayered concept influenced by various factors. Trustworthy and efficient communication helps clients to participate in the decision making process and self management of chronic conditions such as hypertension, heart disease, diabetes, and the like. There are a number of issues that may jeopardize effective and trustworthy communication. This study revealed that factors such as unemployment and/or underemployment could lead to clients' mistrust in health care services. Various research has shown that trust was influenced by unemployment [33,34]. Some other factors encouraged clients to be reluctant about trusting and using health care services. The Iranian community is small and made up of individuals with diverse socioeconomic, political, educational backgrounds. These differences add to the increased complexity in building and maintaining trust. People in this community directly or indirectly know each other, and because of their cultural norms clients are hesitant to reveal their financial limitations and other personal issues. In order to save face and honor, they do not trust health care providers with critical information, nor do they share their issues or follow up with advised treatments. In this study, participants mentioned that clients sometimes looked for shortcut to services and alternatives such as sharing medications, self-treatment and asking family members or friends to bring them medications from Iran to Canada.

Another factor that may cause clients to under-utilize health care services is fear of disclosure. Fear of disclosure was one of the elements that could affect client-provider power relations [35]. Participants in this study mentioned that some of their clients were not comfortable talking about their issues out of fear of deportation. Clients may try to keep some of their issues private in order to save face in front of others within their own community and outside of their own community. In this regard, some participants mentioned that some of their clients skipped providing answers to some questions or deliberately answered them untruthfully because they did not want to reveal private matters. In addition, participants noted that their clients had different understanding of confidentiality. In order to talk about their private lives, they needed to perceive the environment was safe enough to do so. Mistrust of health care services might be a significant barrier to seeking medical care, and following preventive care and treatment regimens. As a result, the health of these clients as well their mental health and well being are at greater risk.

The mental health and well-being of immigrants may be influenced by life-adjustment stressors, socioeconomic isolation and cultural alienation from mainstream society, and may result in somatization issues. One noteworthy finding in this study revealed that most patients sought out treatment from health care providers rather than mental health professionals, when faced with mental health issues, due to cultural stigmas and potential social isolation. Although Iranians understand the importance of mental health, the social stigma surrounding mental illness is considered a major obstacle inhibiting them from seeking psychological treatment. These patients become upset when referred to psychotherapists and they went through a much longer stage of denial, which unfortunately caused delays in seeking help and psychological treatment. Findings showed that social workers played an important role in connecting patients to the right venue of help and throughout the treatment-seeking process.

Over time, immigrants become integrated with main-stream culture, and the longer these clients live in Canada, they realize that alternatives and shortcuts to services are not enough to maintain their health and well being. Many studies have shown that bicultural people and individuals with a high level of integration in their host countries are able to operate effectively in their host country. Although becoming integrated is an ongoing process, it helps one acquire power over one's life world and thus become liberated [36]. In this study, participants believed that integration and biculturalism had a positive impact on Iranian immigrants with regard to becoming self-sufficient in accessing health care services. Health care professional concluded that factors such as the length of residency in Canada, age, language proficiency and level of education contributed positively to the process of immigrant integration and self-sufficiency. These individuals then become a good and reliable source of information and help for newcomers. They play an important role in connecting the community with health care professionals and social workers.

This study revealed intertwined and very complex phenomena. It illustrated that Iranian immigrants experienced many struggles accessing health care services. Health care providers should build a strong relationship with their own communities and provide them basic information about the health care services and system in Canada. The findings of this study suggest that providing information regarding resources and services offers immigrants the opportunity to make their own decisions concerning health care services and to take an active role in their treatment. This study indicates that there is low awareness and understanding of the Canadian health care system and services among immigrants and it is vital that they get the help they need to be able to understand and utilize those services. In addition, health care professionals should communicate with other health providers their success stories in handling patient issues.

Findings indicate that appropriate and acceptable facilities and services play an important role in the way Iranian immigrants' access the health care services they need. As Glouberman stated, "If you look at people's unease about the health care system, it's not because they have found it less than satisfying to use. It has to do with their fear that it won't be there if they need it [37].

## Conclusions

In conclusion, to attain equitable, adequate, and effective access to health care services, immigrants need to be educated and informed about the Canadian health care system and services it provides. It would be of great benefit to this population to hold workshops on health topics and mental health issues, build strong ties with the community, use plain language, create informative and health related websites in both Farsi and English, and provide a Farsi speaking telephone help line to answer their health related issues. These strategies will help immigrants to be empowered and have control over their own lives. Empowerment is still an important key to the issues of adjustment and integration. The more immigrants become integrated the more they use health care services effectively.

Health care providers and social workers concluded that Iranians may not be satisfied with the Canadian health care services due to a lack of knowledge of the system, as well as cultural differences when seeking care, such as fear of disclosure, discrimination, and mistrust of primary care. Therefore, they may put their health at risk by ignoring health issues, delaying care, or using emergency services inappropriately. Consequently, these may impose extra preventable burden on the health care system.

## Limitations

One of the major limitations is that the study group was restricted to Iranian communities in the GTA rather than all Iranian immigrants in Canada. However, although Iranians vary culturally, ethnically, socioeconomically, and linguistically (i.e., Azeri, Kurd, Lour, Pars, Arab, Baluchi, Turkmen, Gilaki etc.), they do share a core of common values and behaviours. Therefore, I believe that to some extent, the result of this study can be applicable to most Iranians living in Canada.

## Competing interests

The authors declare that they have no competing interests.

## Authors' contributions

MD was fully responsible for conducting the study and writing the manuscript.
